# Cerium Oxide-Decorated γ-Fe_2_O_3_ Nanoparticles: Design, Synthesis and *in vivo* Effects on Parameters of Oxidative Stress

**DOI:** 10.3389/fchem.2020.00682

**Published:** 2020-08-04

**Authors:** Maksym Moskvin, Irena Marková, Hana Malínská, Denisa Miklánková, Martina Hüttl, Olena Oliyarnyk, Ognen Pop-Georgievski, Alexander Zhigunov, Eduard Petrovský, Daniel Horák

**Affiliations:** ^1^Institute of Macromolecular Chemistry, Czech Academy of Sciences, Prague, Czechia; ^2^Institute for Clinical and Experimental Medicine, Prague, Czechia; ^3^Institute of Geophysics, Czech Academy of Sciences, Prague, Czechia

**Keywords:** maghemite, cerium oxide, nanoparticles, oxidative stress, antioxidant

## Abstract

Magnetic γ-Fe_2_O_3_/CeO_x_ nanoparticles were obtained by basic coprecipitation/oxidation of iron chlorides with hydrogen peroxide, followed by precipitation of Ce(NO_3_)_3_ with ammonia. The appearance of CeO_x_ on the magnetic particle surface was confirmed by X-ray photoelectron spectroscopy (XPS), powder X-ray diffraction (XRD), and elemental analysis; a magnetometer was used to measure the magnetic properties of γ-Fe_2_O_3_/CeO_x_. The relatively high saturation magnetization of the particles (41.1 A·m^2^/kg) enabled magnetic separation. The surface of γ-Fe_2_O_3_/CeO_x_ particles was functionalized with PEG-neridronate of two different molecular weights to ensure colloidal stability and biocompatibility. The ability of the particles to affect oxidative stress in hereditary hypertriglyceridemic (HHTg) rats was tested by biological assay of the liver, kidney cortex, and brain tissues. An improvement was observed in both enzymatic [superoxide dismutase (SOD), catalase (CAT), and glutathione peroxidase (GPx)] and non-enzymatic (reduced (GSH) and oxidized (GSSG) glutathione) levels of antioxidant defense and lipid peroxidation parameters [4-hydroxynonenal (4-HNE) and malondialdehyde (MDA)]. The results corresponded with chemical determination of antioxidant activity based on 2,2-diphenyl-1-picrylhydrazyl (DPPH) assay, proving that in the animal model γ-Fe_2_O_3_/CeO_x_@PEG_2,000_ nanoparticles effectively scavenged radicals due to the presence of cerium oxide, in turn decreasing oxidative stress. These particles may therefore have the potential to reduce disorders associated with oxidative stress and inflammation.

## Introduction

On a cellular level, many types of inflammation are associated with the excessive formation of reactive oxygen species (ROS) such as superoxide/hydroxyl radicals and hydrogen peroxide (H_2_O_2_) (Schieber and Chandel, 2014). Their ability to be scavenged is important in combating serious oxidative stress-related diseases, such as atherosclerosis, liver steatosis, diabetes mellitus, cancer, Alzheimer's disease, and aging. All these disorders are associated with severe damage to lipids, proteins, and nucleic acids (Poprac et al., 2017; Rehman and Akash, 2017). Due to ability of cerium oxide (CeO_x_) nanoparticles to mimic antioxidant enzymes such as superoxide dismutase (SOD) (Korsvik et al., 2007; Celardo et al., 2011), catalase (CAT) (Pirmohamed et al., 2010), and peroxidase (Jiao et al., 2012), they have recently received considerable attention as a potentially useful antioxidant agent in mitigating these diseases. These nanoparticles are adept at scavenging almost all types of reactive species, including hydroxyl and nitroxyl radicals. The impressive radical scavenging activity of CeO_x_, which can take the form of CeO_2_ or Ce_2_O_3_, is attributed to mutations in the oxidation state between Ce^4+^ and Ce^3+^ ions (Asati et al., 2009; Xu and Qu, 2014). In order to enhance scavenging activity, the surface area of CeO_x_ must be increased, achieved by decreasing their overall size to a nanometer scale.

In addition to their usefulness in biomedicine, drug delivery, and analysis, CeO_x_ nanoparticles are also utilized in numerous engineering applications, such as solid-oxide fuel and solar cells (Stambouli and Traversa, 2002; Corma et al., 2004), corrosion protection (Ivanov et al., 2009), and catalysis (Walkey et al., 2015). The many approaches to preparing CeO_x_ nanoparticles largely depend on variations in morphology, size, and the tendency to aggregate. These include ball milling, thermal decomposition, precipitation, spray pyrolysis, hydro-/solvothermal or sol-gel methods, surfactant-assisted techniques, and green synthesis using natural stabilizers (Xu and Qu, 2014). Precursors of CeO_x_ synthesis include ammonium cerium nitrate, cerium sulfate, nitrate, and chloride. However, a key role in the fabrication of CeO_x_ nanoparticles is played by surface engineering, which ensures colloidal stability in physiological media, biocompatibility, and/or functionalization with reactive groups employed for further conjugation of biomolecules, targeting peptides, and/or drugs. Among the conventionally used coatings are dextran (Karakoti et al., 2007; Weaver and Stabler, 2015), polyethylenimin (Lee et al., 2013), poly(ethylene glycol) (Karakoti et al., 2009), poly(acrylic acid) (Wu et al., 2018), cyclodextrin (Xu et al., 2013), and glucose (Li et al., 2013).

Another class of inorganic particles attracting growing attention in biomedicine is the iron oxides (magnetite Fe_3_O_4_ and maghemite γ-Fe_2_O_3_). These materials are superparamagnetic, magnetizing in the presence of an external magnetic field; however, they exhibit no residual magnetization in the absence of a magnetic field (Li et al., 2017). These properties enable the targeting tissues required by drug delivery systems and/or monitoring of iron oxide-labeled cells using non-invasive *in vivo* magnetic resonance imaging (MRI). Moreover, magnetic nanoparticles can control the movement and transportation of biomolecules and/or generate heat in an external magnetic field to kill tumor cells (Jordan et al., 1993). A major advantage of Fe_3_O_4_ and γ-Fe_2_O_3_ is their biocompatibility, with ferumoxytol (Feraheme) approved by the US Food and Drug Administration in 2009 for the treatment of patients with chronic kidney disease suffering from iron deficiency (Toth et al., 2017).

The aim of this report was to merge both the magnetic and antioxidant properties of the maghemite core and cerium oxide shell into a new delivery system that would be colloidally stable and magnetically manipulable. Antioxidant properties were verified based on chemical and biological *in vivo* tests of using a rat model of metabolic syndrome and prediabetes—the hereditary hypertriglyceridaemic rat (Malinska et al., 2019). Effect of cerium oxide-decorated γ-Fe_2_O_3_ nanoparticles on oxidative stress and antioxidant defense parameters has been evaluated.

## Experimental

### Materials

FeCl_2_ · 4H_2_O, FeCl_3_ · 6H_2_O, Ce(NO_3_)_3_ · 6H_2_O, trisodium citrate dihydrate, and 2,2-diphenyl-1-picrylhydrazyl (DPPH) were purchased from Sigma-Aldrich (St. Louis, MO, USA). NH_4_OH (25 wt.%), HCl (35 wt.%), H_2_O_2_ (30 wt.%), ethanol (96 %), propan-2-ol, and all other chemicals were purchased from LachNer (Neratovice, Czechia). Poly(ethylene glycol)-neridronate (PEG-Ner) was synthesized using PEG at a molecular weight of 2,000 or 5,000 Da as described previously (Kostiv et al., 2017).

### Preparation of Cerium Oxide-Decorated Maghemite Nanoparticles (γ-Fe_2_O_3_/CeO_x_)

γ-Fe_2_O_3_ cores were obtained by modifying a previously published procedure (Moskvin and Horák, 2016). Briefly, an aqueous FeCl_3_ and FeCl_2_ solution (500 ml; FeCl_3_/FeCl_2_ = 2/1 mol/mol) was precipitated with NH_4_OH (20 ml; pH 11) at 70°C by stirring (700 rpm); the resulting magnetite (Fe_3_O_4_) was oxidized by the addition of H_2_O_2_ (5 ml) and 35 % HCl (~12 ml) at 90°C. The final γ-Fe_2_O_3_ particles were washed with water five times using a magnetic separation and sonication (Hielscher UP-400St; Teltow, Germany; 20 W and 70 % amplitude) for 10 min and stabilized with trisodium citrate (60 mg per 300 mg of γ-Fe_2_O_3_). The γ-Fe_2_O_3_ surface was then modified by dispersion precipitation of cerium(III) nitrate (300 mg; γ-Fe_2_O_3_/Ce(NO_3_)_3_ = 1/1 w/w) with NH_4_OH (1 ml; pH 11) in a mixture of water/propan-2-ol = 1/1 v/v (25 ml) at 60°C for 3 h. Subsequently, the colloid was thoroughly washed with water 10 times (20 ml each) under sonication and magnetic separation and stabilized by the addition of aqueous trisodium citrate as described above; the final concentration was 4 mg of γ-Fe_2_O_3_/CeO_x_ per ml (pH ~8).

Pure CeO_x_ particles used as a reference material in DPPH assay were prepared as described above, but in the absence of γ-Fe_2_O_3_.

### PEGylation of Cerium Oxide-Decorated Maghemite Nanoparticles (γ-Fe_2_O_3_/CeO_x_@PEG)

Aqueous γ-Fe_2_O_3_/CeO_x_ (100 mg) dispersion (10 ml) was added to a solution of poly(ethylene glycol)-neridronate (40 mg) in water (15 ml). The reaction proceeded at room temperature (RT) for 1 h with sonication for 5 min. The resulting γ-Fe_2_O_3_/CeO_x_@PEG_2,000_ and γ-Fe_2_O_3_/CeO_x_@PEG_5,000_ nanoparticles were washed with water (100 ml).

### Particle Characterization

γ-Fe_2_O_3_ nanoparticles were visualized using the Tecnai G2 Spirit transmission electron microscope (TEM; FEI; Brno, Czechia). Number-average diameter (*D*_n_ = Σ*D*_i_/N, where *D*_i_ is the diameter of the i-th particle and N is the total number of particles), weight-average diameter (*D*_w_ = Σ$D_{\text{i}}^{4}$/Σ$D_{\text{i}}^{3}$) and dispersity (Ð = *D*_w_/*D*_n_) were calculated from at least 300 individual particles on the micrographs using Atlas software (Tescan Digital Microscopy Imaging; Brno, Czechia). Surface ζ-potential, hydrodynamic diameter *D*_h_, and polydispersity *PI* were measured by dynamic light scattering (DLS) using a ZEN3600 Nano-ZS ZetaSizer (Malvern Instruments; Malvern, Worcestershire, UK).

The X-ray diffraction (XRD) patterns of dry γ-Fe_2_O_3_, γ-Fe_2_O_3_/CeO_x_, and γ-Fe_2_O_3_/CeO_x_@PEG_2,000_ particles were measured in reflection mode using the Explorer high-resolution diffractometer (GNR Analytical Instruments; Agrate Conturbia, Italy) equipped with the Mythen 1K one-dimensional silicon strip detector (Dectris; Baden, Switzerland). Radiation of MoKα (λ = 0.7107 Å) monochromatized with Zr foil (β-filter) was used for diffraction in the range of 2θ = 2–40° with step 0.1° and exposure time 10 s. FTIR spectra were recorded in KBr pellet on a Bruker IFS 55 spectrometer (Billerica, MA, USA) with a DTGS detector under resolution of 4 cm^−1^ and 32 scans. Cerium, iron, carbon, hydrogen and nitrogen content was determined using the Perkin-Elmer 3110 atomic absorption spectrometer after mineralizing particles with HCl (1:1) at 80°C and the Perkin-Elmer 2400 CHN elemental analyzer. Magnetic properties of the particles were measured using the EV9 vibrating sample magnetometer (DSM Magnetics ADE; Lowell, MA, USA) at RT; magnetization was corrected for paramagnetic contribution in fields >400 kA/m.

### X-ray Photoelectron Spectroscopy (XPS)

XPS was performed using a K-Alpha^+^ XPS spectrometer (Thermo Fisher Scientific; Loughborough, UK) operating at a base pressure of 1.0 × 10^−7^ Pa. Data were acquired and processed using Thermo Scientific Avantage software. γ-Fe_2_O_3_, γ-Fe_2_O_3_/CeO_x_, and γ-Fe_2_O_3_/CeO_x_@PEG_2,000_ nanoparticles were uniformly deposited on clean silicon substrates and analyzed using Al Kα X-ray microfocused monochromated radiation (400 μm spot size) with a pass energy of 200 and 50 eV for survey and high-energy resolution core-level spectra, respectively. The X-ray angle of incidence was 30°, with the emission angle normal to the surface. The K-Alpha dual-charge compensation system was employed during analysis, using electrons and low-energy argon ions to prevent any localized charge build-up. The acquisition times were kept <1 min to avoid the reduction of cerium oxide after the exposure to XPS radiation under high vacuum (Zhang et al., 2004). The high-resolution spectra were fitted using Voigt profiles and referenced to the C 1 s peak attributed to C-C and C-H at 285.0 eV binding energy, which was controlled using the standard photoelectron peak positions for poly(ethylene terephthalate), Cu, Ag, and Au. Atomic concentrations of phosphorous, carbon, nitrogen, oxygen, iron, and cerium were determined from the P 2p, C 1s, N 1s, O 1s, Fe 2p, and Ce 3d photoelectron peak areas after Shirley inelastic background subtraction. The amount of cerium in oxidation states Ce^3+^ and Ce^4+^ was approximated by comparing the measured spectra with those of Ce_2_O_3_ and CeO_2_ taken from Avantage database.

### DPPH Radical Scavenging Assay

The antioxidant activity of the particles was measured colorimetrically by 2,2-diphenyl-l-picrylhydrazyl (DPPH) assay. Briefly, 0.1 mM ethanolic DPPH solution (0.6 ml) was added in a 2 ml Eppendorf tube to a mixture of ethanol (1.2 ml) and aqueous particle dispersion (0.2 ml; 4.4 mg of γ-Fe_2_O_3_/ml) with shaking for 1 min using a Vortex shaker (1,000 rpm), leaving the reaction to continue in darkness for 30 min. The above mixture was used as a control or blank solution in the absence of the particles or DPPH, respectively. Absorbance of the solution was measured by the Specord 250 Plus UV-Vis spectrophotometer (Analytik Jena, Germany) against ethanol at 517 nm. Antioxidant activity (*AA*) was calculated using Equation (1):

(1) $$\begin{array}{l} \text{AA}=\frac{A_{c}-A_{p}+A_{b}}{A_{c}}\cdot 100 \end{array}$$

where *A*_c_, *A*_p_, and *A*_b_ is the absorption of control, particle dispersion, and blank, respectively.

### Administration of γ-Fe_2_O_3_/CeO_x_ Nanoparticles Using an Experimental Animal Model

To investigate the *in vivo* effect of cerium oxide nanoparticles, a non-obese rat model of metabolic syndrome and prediabetes was used. The hereditary hypertriglyceridemic (HHTg) strain of rat exhibits dyslipidemia, tissue resistances to insulin action, fatty liver, mild hypertension, and low-grade chronic inflammation in the absence of obesity. Animals were fed a standard diet and given free access to food and water. Five-month-old male rats were randomly divided into five experimental groups of eight animals; untreated rats, treated with γ-Fe_2_O_3_ (control), γ-Fe_2_O_3_/CeO_x_, γ-Fe_2_O_3_/CeO_x_@PEG_2,000_, and γ-Fe_2_O_3_/CeO_x_@PEG_5,000_ nanoparticles. Nanoparticle dispersions (4 mg/ml) were administrated i.v. to the *vena caudalis* at a dose of 0.5 ml, untreated rats were given i.v. 0.5 ml saline instead of nanoparticle dispersion. Three days after nanoparticle application, the animals were sacrificed in a postprandial state. The collected tissue samples (liver, kidney cortex and brain) were immediately frozen in liquid nitrogen and stored at −80°C before analysis.

All experiments were performed in agreement with the Animal Protection Law of the Czech Republic (311/1997) in compliance with European Community Council recommendations (86-609/ECC) for the use of laboratory animals and approved by the Ethics Committee of the Institute for Clinical and Experimental Medicine, Prague.

### Basal Metabolic Analysis

Serum levels of triglycerides, glucose, and total cholesterol were measured using commercially available kits (Erba Lachema; Brno, Czechia). Alanine aminotransferase (ALT) and aspartate aminotransferase (AST) enzyme activity was determined spectrophotometrically using routine clinical biochemistry methods and Roche Diagnostics kits (Mannheim, Germany).

### Oxidative Stress Parameters

Levels of reduced (GSH) and oxidized (GSSG) forms of glutathione were determined by high-performance liquid chromatography (HPLC) with fluorescent detection using a HPLC diagnostic kit (Chromsystems; Gräfelfing, Germany).

The antioxidant enzyme activity of superoxide dismutase (SOD), catalase (CAT), and glutathione peroxidase (GPx) was measured using commercially available kits (Sigma-Aldrich and Cayman Chemicals; Ann Arbor, MI, USA). Malondialdehyde (MDA), a parameter of lipid peroxidation, was determined by HPLC with fluorescence detection; 4-hydroxynonenal (4-HNE), a sensitive product of lipid peroxidation, was detected by rat ELISA assay (MyBiosource; San Diego, CA, USA).

### Statistical Analysis

All data were expressed as mean ± SEM. Statistical analysis was performed by one-way ANOVA with Fisher's *post-hoc* test using Statistica 12 software. Group comparisons were made against the control group of γ-Fe_2_O_3_-administered animals. Statistical significance was defined as *p* < 0.05.

## Results

### γ-Fe_2_O_3_ Nanoparticles

**Figure 1:**
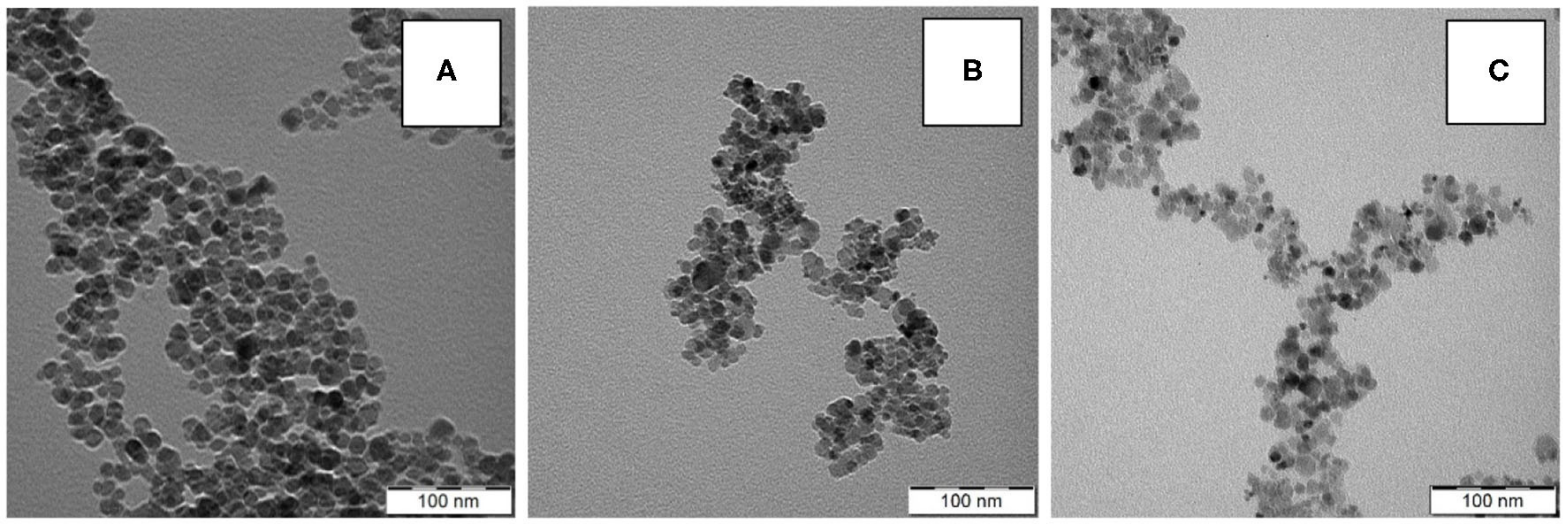
TEM micrographs of **(A)** γ-Fe_2_O_3_, **(B)** γ-Fe_2_O_3_/CeO_x_, and **(C)** γ-Fe_2_O_3_/CeO_x_@PEG_2,000_ nanoparticles.

Starting iron oxide particles were prepared using a one-pot procedure consisting of two steps: (*i*) aqueous precipitation of Fe(II) and Fe(III) chlorides with ammonia, followed by (*ii*) oxidation with H_2_O_2_ and electrostatic stabilization achieved by the addition of trisodium citrate. The morphology of particles was almost spherical, with a mean diameter *D*_n_ = 15 nm and a dispersity Ð = 1.28 according to TEM, indicating moderately broad particle size distribution (Figure 1A; Table 1). These results were in agreement with DLS analysis, which documented polydispersity *PI* = 0.21, hydrodynamic size *D*_h_ = 68 nm, and ζ-potential = −43 mV due to citrate stabilization. *D*_h_ proved larger than *D*_n_ because the former is a z-average diameter affected by the presence of larger particles to a much greater extent than the latter diameter of dry particles determined by TEM. Moreover, the particles had a tendency to slight aggregation in water.

**Table 1:** Characterization of nanoparticles.

**Particles**	***D*_**n**_ (nm)**	**Ð**	***D*_**h**_ (nm)**	***PI***	**ζ-potential (mV)**	***AA* (%)**
γ-Fe_2_O_3_	15	1.28	68	0.21	−43	13
γ-Fe_2_O_3_/CeO_x_	16 (6)^a^	1.33	85	0.27	−45	78
γ-Fe_2_O_3_/CeO_x_@PEG_2,000_	16 (6)^a^	1.39	63	0.20	−29	89
γ-Fe_2_O_3_/CeO_x_@PEG_5,000_	17 (6)^a^	1.37	71	0.22	−15	85

a *CeO_x_ nanoparticles; D_n_, number-average particle diameter (TEM); Ð, dispersity (TEM); D_h_, hydrodynamic diameter (DLS); PI, polydispersity (DLS); AA, antioxidant activity*.

**Figure 2:**
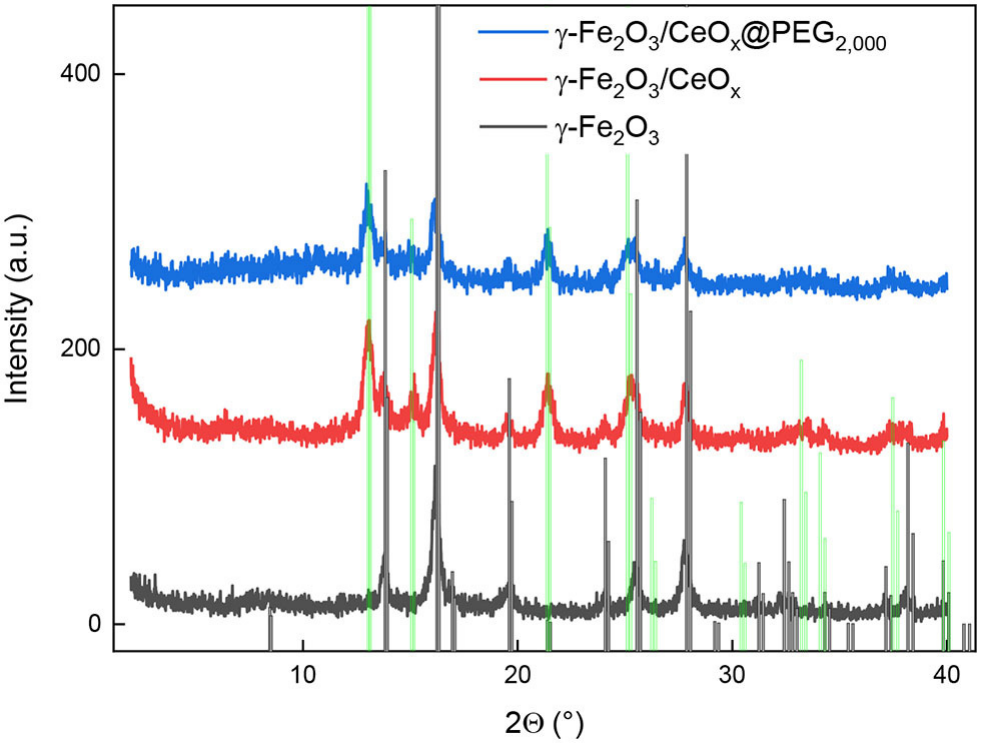
Diffraction pattern of γ-Fe_2_O_3_ (black), γ-Fe_2_O_3_/CeO_x_ (red), and γ-Fe_2_O_3_/CeO_x_@PEG_2,000_ nanoparticles (blue) compared to that of γ-Fe_2_O_3_ and CeO_2_ from the Crystallography Open Database (vertical black and green lines, respectively).

To determine whether magnetite (Fe_3_O_4_) or maghemite (γ-Fe_2_O_3_) was obtained during coprecipitation, an XRD diffractogram of the synthesized iron oxide particles was compared with the reference diffraction lines of cubic γ-Fe_2_O_3_ according to the Crystallography Open Database (COD)^1^; COD ID 96-900-6317 (Figure 2; reference vertical diffraction lines were marked in black). The most intense peaks for Fe_3_O_4_ and γ-Fe_2_O_3_ coincided; the differences in peak positions were minor, but clearly visible, suggesting that the particles contained γ-Fe_2_O_3_. The less intense γ-Fe_2_O_3_ peak, at 2θ = 22.49°, was observed as a small bump in the diffractogram. The particle sizes *L* were estimated using the Scherrer (Equation 2):

(2) $$\begin{array}{l} L=\frac{K\lambda }{\beta \cdot \text{ cos }\Theta } \end{array}$$

where K is the shape factor (usually 0.9) and β is the full width at half maximum (FWHM) of reflection. For this purpose, the most intense peak at 2θ = 16.24° was chosen, possessing FWHM = 0.4734°. Assuming that the particle is fully crystalline, average size of 17.4 nm was obtained, which agrees well with TEM results.

### Cerium Oxide-Decorated γ-Fe_2_O_3_ Nanoparticles (γ-Fe_2_O_3_/CeO_x_)

A TEM micrograph of γ-Fe_2_O_3_/CeO_x_ particles could not distinguish CeO_x_ from γ-Fe_2_O_3_ due to their similar electron densities (Figure 1B); however, rather small CeO_x_ nanoparticles (~6 nm) were clearly visible in the micrograph in addition to the pure γ-Fe_2_O_3_ particles (Figure 1A). Neither the dispersity (Ð = 1.33) nor morphology of the γ-Fe_2_O_3_/CeO_x_ particles (Figure 1B) substantially differed from those of starting γ-Fe_2_O_3_. This was in agreement with results obtained on magnetic cerium oxide nanoconjugates prepared by glutaraldehyde-crosslinked polyethyleneimine-coated mixture of magnetic and cerium oxide nanoparticles (Turin-Moleavin et al., 2019). Compared to the γ-Fe_2_O_3_ particles, the hydrodynamic size of γ-Fe_2_O_3_/CeO_x_ nanoparticles in water increased (*D*_h_ = 85 nm), probably due to the presence of CeO_x_ and the citrate layer; ζ-potential did not substantially change from that of γ-Fe_2_O_3_.

From the XRD spectrum of γ-Fe_2_O_3_/CeO_x_ nanoparticles, it was evident that CeO_2_ was present in the composite in its crystalline form (Figure 2). The conclusion was made by comparing the diffractogram with COD ID 96-434-3162. Therefore, the diffraction curve, corresponding to the γ-Fe_2_O_3_/CeO_x_ composite (Figure 2, red line), showed the superposition of neat crystalline phases of γ-Fe_2_O_3_ and CeO_2_.

**Figure 3:**
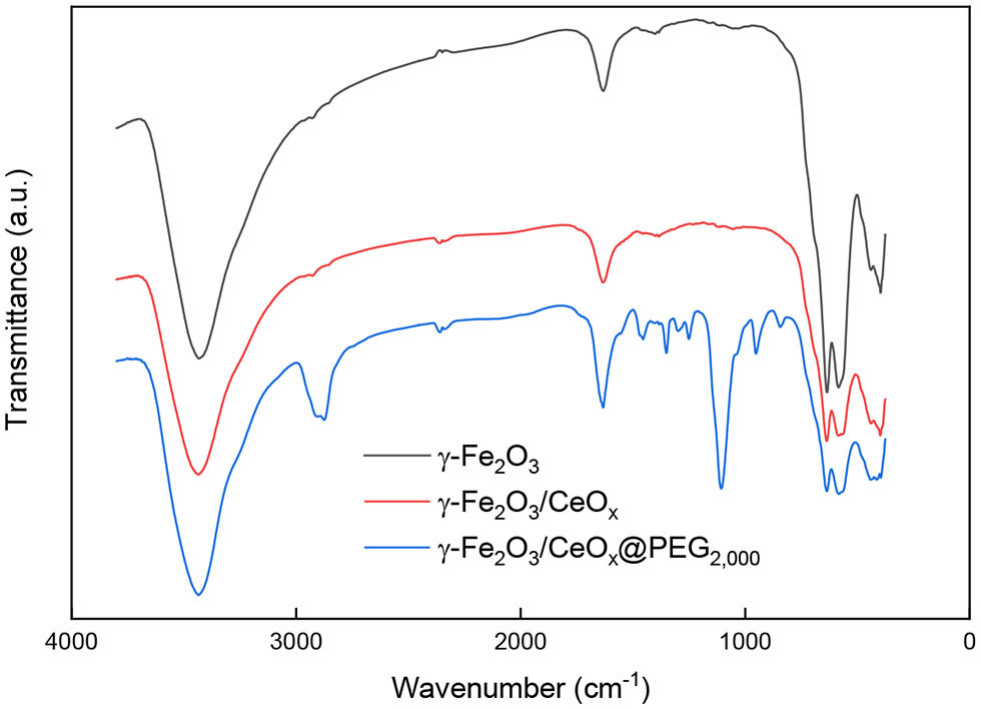
FTIR spectra of γ-Fe_2_O_3_, γ-Fe_2_O_3_/CeO_x_, and γ-Fe_2_O_3_/CeO_x_@PEG_2,000_ nanoparticles.

FTIR spectra of the γ-Fe_2_O_3_ and γ-Fe_2_O_3_/CeO_x_ particles were recorded to probe their surface structure (Figure 3); the spectra did not substantially differ, with the exception of high intensities of γ-Fe_2_O_3_ bands in the region of 400–800 cm^−1^, corresponding to the vibrations of atoms in the crystalline lattice of iron oxide. A broad band of OH- stretching vibrations was situated at ~3,400 cm^−1^ and ascribed to hydroxyl groups on the particle surface and partially to water adsorbed by hygroscopic KBr used as a binder for the sample preparation.

**Figure 4:**
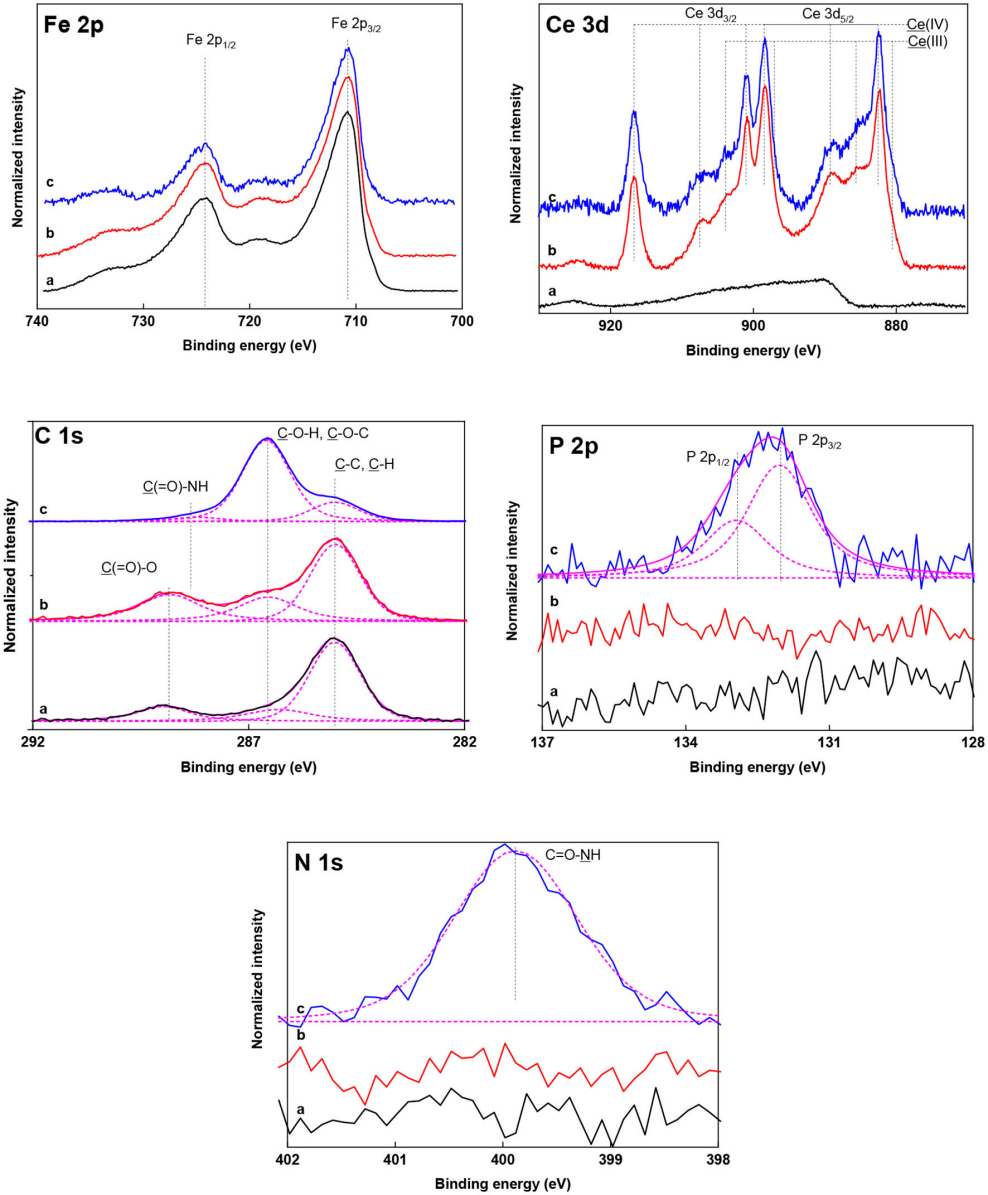
High-resolution Fe 2p, Ce 3d, C 1s, P 2p, and N 1s XPS spectra of (a) γ-Fe_2_O_3_, (b) γ-Fe_2_O_3_/CeO_x_, and (c) γ-Fe_2_O_3_/CeO_x_@PEG_2,000_ nanoparticles. Spectra and their resulting fitted envelopes are presented with full and magenta lines, respectively. Individual contributions of the fitted curve are presented with dashed lines.

The covalent structure of the nanoparticles was further investigated after each modification step by XPS (Figure 4; Table 2). The pure γ-Fe_2_O_3_ nanoparticles exhibited a characteristic Fe 2p spectrum previously reported for maghemite (Zasonska et al., 2016; Shatan et al., 2019). Successful deposition of cerium oxide on γ-Fe_2_O_3_ was verified in the high-resolution Ce 3d spectra of γ-Fe_2_O_3_/CeO_x_ nanoparticles based on the appearance of characteristic Ce 3d_5/2_-Ce 3d_3/2_ spin-orbital multiplet splitting. Ce content reached ~26 wt.%, while iron content in the γ-Fe_2_O_3_/CeO_x_ particles dropped to 38.7 ± 1.7 wt.% from 64.5 ± 2.2 wt.% in γ-Fe_2_O_3_. The Ce^4+^/Ce^3+^ ratio was determined to be 7.8 (Table 2), showing that cerium was predominantly in the form of CeO_2_ (~23.1 wt.%). Small peaks were found in the C 1s spectra of pure γ-Fe_2_O_3_ and γ-Fe_2_O_3_/CeO_x_ nanoparticles, originating from organic molecules adsorbed on the nanoparticle surface during its exposure to air.

**Table 2:** Surface composition of γ-Fe_2_O_3_, γ-Fe_2_O_3_/CeO_x_, and γ-Fe_2_O_3_/CeO_x_@PEG_2,000_ nanoparticles determined by XPS analysis.

**Electronic state of element**	**γ-Fe_**2**_O_**3**_(wt.%)**	**γ-Fe_**2**_O_**3**_/CeO_**x**_(wt.%)**	**γ-Fe_**2**_O_**3**_/CeO_**x**_@PEG_**2, 000**_(wt.%)**
P 2p	–^a^	–	1.8 ± 0.1
C 1s	7.5 ± 1.9	8.8 ± 1.2	34.0 ± 0.5 26.1 ± 0.1^b^
N 1s	–	–	0.9 ± 0.1
O 1s	28.0 ± 0.4	26.2 ± 0.8	36.2 ± 0.2
Fe 2p	64.5 ± 2.2	38.7 ± 1.4	13.9 ± 0.2
Ce 3d	–	26.3 ± 0.7	13.2 ± 0.4
Ce^4+^/Ce^3+^	–	7.8 ± 0.1	7.7 ± 0.2

a *Below the detection limit of XPS measurement*;

b *content of PEG according to C-O-C bond*.

**Figure 5:**
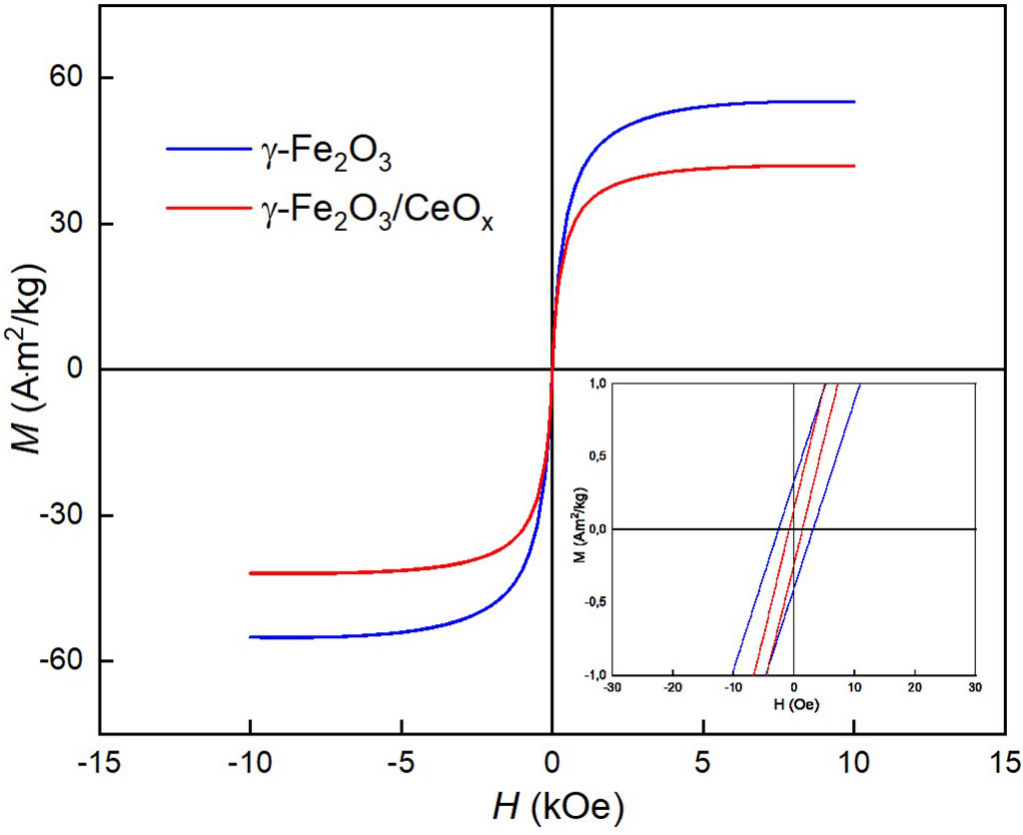
Magnetization hysteresis loop of γ-Fe_2_O_3_ (blue) and γ-Fe_2_O_3_/CeO_x_ nanoparticles (red) at room temperature; magnified loop is inserted. *H*, magnetic field; *M*, magnetization.

Magnetic measurements of γ-Fe_2_O_3_ and γ-Fe_2_O_3_/CeO_x_ nanoparticles revealed saturation magnetization values of *M*_s_ = 53.3 and 41.1 A·m^2^/kg, respectively (Figure 5). This means that ~23 wt.% of CeO_x_ was present in the γ-Fe_2_O_3_/CeO_x_ particles, as calculated from the dependence of *M*_s_ on γ-Fe_2_O_3_ content. Remanent magnetization and coercivity of γ-Fe_2_O_3_ and γ-Fe_2_O_3_/CeO_x_ were very low (*M*_rs_ = 0.4 and 0.2 A·m^2^/kg and *H*_c_ = 2.8 and 1.1 Oe, respectively), as determined using interpolation between the neighboring data points on the hysteresis loop. This shows the absence of a domain structure due to the collective behavior and high concentration of superparamagnetic particles. As a result, the particles remained dispersible in water in the absence of an external magnetic field and easily magnetized and manipulated in its presence, thus enabling magnetic separation of the γ-Fe_2_O_3_/CeO_x_ particles.

### γ-Fe_2_O_3_/CeO_x_@PEG Nanoparticles

**Figure 6:**
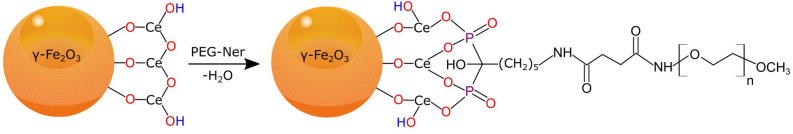
Synthesis of γ-Fe_2_O_3_/CeO_x_@PEG nanoparticles using PEG-neridronate.

To provide efficient steric stabilization even in buffered media at a high ionic strength and to enhance biocompatibility of the γ-Fe_2_O_3_/CeO_x_ nanoparticles, their surfaces were modified with PEG-neridronate (PEG-Ner) at two different molecular weights: *M*_w_ = 2,000 and 5,000 Da (Figure 6). PEG-Ner was obtained from sodium neridronate and *O*-[(*N*-succinimidyl)succinylaminoethyl]-*O*'-methylpoly(ethylene glycol) as previously described (Kostiv et al., 2017). As PEG <5 kDa is typically used for PEGylation of particles in *in vivo* studies (Zalipsky, 1995), γ-Fe_2_O_3_/CeO_x_@PEG_2,000_ particles were investigated by TEM, XPS, FTIR, and elemental analysis. According to TEM, γ-Fe_2_O_3_/CeO_x_@PEG_2,000_ nanoparticles resembled to γ-Fe_2_O_3_/CeO_x_, with PEG coating invisible due to its low electron density (Figure 1C). The γ-Fe_2_O_3_/CeO_x_@PEG_2,000_ particles had a number-average diameter *D*_n_ = 15 and 6 nm for maghemite and cerium oxide particles, respectively; dispersity Ð = 1.39 was higher than that of pure γ-Fe_2_O_3_ nanoparticles due to the presence of tiny CeO_x_ particles; the hydrodynamic diameter of the γ-Fe_2_O_3_/CeO_x_@PEG_2,000_ and γ-Fe_2_O_3_/CeO_x_@PEG_5,000_ nanoparticles reached *D*_h_ = 63 and 71 nm, respectively, with *PI* = 0.2 (Table 1). The PEG-modified particles were smaller in size, as they have a reduced tendency to aggregate due to improved steric stabilization. Moreover, the effect of the counter-ion layer on citrate-stabilized γ-Fe_2_O_3_/CeO_x_ particles may have caused them to increase in size. Absolute value of ζ-potential of PEGylated γ-Fe_2_O_3_/CeO_x_ particles decreased due to the presence of electroneutral PEG shell; nevertheless, it was still negative (Table 1).

X-ray diffraction spectrum of γ-Fe_2_O_3_/CeO_x_@PEG_2,000_ nanoparticles was presented in Figure 2 as a blue curve. Peak positions and relative intensities of γ-Fe_2_O_3_ and CeO_2_ phases were the same as for the γ-Fe_2_O_3_/CeO_x_, confirming no changes in crystalline structure of the two components. Despite the fact that the content of PEG_2,000_ was relatively high (>26 wt.% according to XPS), no crystalline peak corresponding to PEG was observed. Moreover, there was a wide peak with a mean value of 2θ = 12.3°, which could be ascribed to the mean distance between PEG chains.

FTIR spectrum of γ-Fe_2_O_3_/CeO_x_@PEG_2,000_ nanoparticles had strong valence vibration band of amide N-H bonds at 1,550 cm^−1^, corresponding to PEG-neridronate linker (Figure 3). Bands at 2,870, 1,467, and 1,342 cm^−1^ were ascribed to PEG aliphatic chain vibrations of CH_3_ and scissor and wagging vibrations of CH_2_, respectively. Vibrations of C-O-C groups of PEG were observed at 1,240, 1,097, and 960 cm^−1^. Band at 1,643 and 1,278 cm^−1^ was attributed to vibrations of amide carbonyls and P=O bonds originating from phosphonic acid residues of neridronate. This confirmed successful anchoring of PEG-Ner to the particle surface.

XPS spectroscopy confirmed the successful chelation of PEG-Ner to the cerium oxide shell. The strong binding of PEG_2,000_-Ner to γ-Fe_2_O_3_/CeO_x_ nanoparticles was clearly evident from high-resolution P 2p, C 1s, and N 1s spectra of γ-Fe_2_O_3_/CeO_x_@PEG_2,000_ particles (Figure 4). The presence of neridronate was detected based on its characteristic P 2p and N 1s signals from the bisphosphonate and amide groups at 132.1 and 399.8 eV, respectively. The experimentally found bisphosphonate/amide ratio (0.92 mol/mol) corresponded with the theoretical ratio in PEG-Ner that equaled one. PEG binding to the cerium oxide shell induced a significant increase of C 1s content to 34.0 ± 0.5 wt.% (Table 2). In addition, the C 1s spectrum of the γ-Fe_2_O_3_/CeO_x_@PEG_2,000_ particles was dominated by a characteristic PEG C–O–C signal at 286.6 eV (Pop-Georgievski et al., 2018); PEG reached a quantity of 26.1 ± 0.1 wt.%. Coating the γ-Fe_2_O_3_/CeO_x_ with PEG induced a drop in iron and cerium content to 13.9 ± 0.2 and 13.2 ± 0.4 wt.%, respectively, further confirming the successful surface modification of the γ-Fe_2_O_3_/CeO_x_ particles with PEG. Modification of the particles with PEG did not significantly reduce the Ce^4+^/Ce^3+^ ratio amounting to 7.7 (Table 2). This result confirmed that the γ-Fe_2_O_3_/CeO_x_ possessed high CeO_2_ content (~11.7 wt.%) even after the reaction with bisphosphonate groups of PEG-Ner.

Atomic absorption spectroscopy and elemental analysis were used as further methods to characterize the elemental composition of particles (Table 3). Experimentally found Fe, Ce, C, and N content was close to theoretical values. The percentage of analyzed Ce (20.1 wt.%) almost corresponded to Ce content (19 wt.%) based on magnetization measurement and assuming CeO_2_ to be a shell. The Fe content found in γ-Fe_2_O_3_/CeO_x_ nanoparticles (54.2 wt.%) was slightly higher than theoretical value (50 wt.%), probably due to the moderate removal of cerium oxide during particle purification. Carbon analysis revealed 12.7 wt.% of C, indicating that the γ-Fe_2_O_3_/CeO_x_@PEG_2,000_ particles contained 25.4 wt.% of PEG, which means that 63 wt.% of PEG loaded in the reaction mixture was actually attached to the particle surface. Nitrogen content originating from the neridronate groups anchored to the iron oxide surface amounted to 0.5 wt.% (Table 3). The discrepancies between elemental analysis and XPS can be attributed to the latter technique only accounting for the particle surface layer and not the particle bulk.

**Table 3:** Elemental composition of γ-Fe_2_O_3_/CeO_x_ and γ-Fe_2_O_3_/CeO_x_@PEG_2,000_ nanoparticles.

**Element particles**	**Fe (wt.%)^a^**		**Ce (wt.%)^a^**		**C (wt.%)**		**H (wt.%)**		**N (wt.%)**	
	**Th**.	**Exp**.	**Th**.	**Exp**.	**Th**.	**Exp**.	**Th**.	**Exp**.	**Th**.	**Exp**.
Fe_2_O_3_/CeO_x_	50	54.2	23.3	20.1	–	–	–	–	–	–
γ-Fe_2_O_3_/CeO_x_@PEG_2,000_	35.6	39.5	16.6	14.3	14.4	12.7	2.4	3.2	0.3	0.5

a *Based on atomic absorption spectroscopy; Th., theoretical content, Exp., experimentally found content*.

### ROS Scavenging by CeO_x_ and γ-Fe_2_O_3_/CeO_x_@PEG Nanoparticles

The antioxidant activity (*AA*) of CeO_x_ particles determined by DPPH assay reached 96%, while that of γ-Fe_2_O_3_/CeO_x_ nanoparticles was 78%, which was a much higher percentage than that of pure γ-Fe_2_O_3_ at only 13% (Table 1). Both γ-Fe_2_O_3_/CeO_x_@PEG_2,000_ and γ-Fe_2_O_3_/CeO_x_@PEG_5,000_ nanoparticles exhibited similar antioxidant activity, reaching 89 and 85%, respectively. The increase in *AA*, compared to that of γ-Fe_2_O_3_/CeO_x_ particles, can be ascribed to the enhanced surface area of the PEG-modified particles available for scavenging as a result of reduced particle aggregation. A small decrease of antioxidant activity of PEGylated γ-Fe_2_O_3_/CeO_x_ particles compared to that of pure CeO_x_ can be ascribed to the presence of iron oxide with reduced *AA*.

### *In vivo* Effect of γ-Fe_2_O_3_/CeO_x_@PEG Nanoparticles on Oxidative Stress in HHTg Rats

Through biological experiments, effect of γ-Fe_2_O_3_, γ-Fe_2_O_3_/CeO_x_, and γ-Fe_2_O_3_/CeO_x_@PEG nanoparticles on oxidative stress and the antioxidant defense in HHTg rats was investigated. Preliminarily, various exposure times were tested (1, 3, and 5 days), with the greatest nanoparticle effect found after 3 days of exposure. Nanoparticle administration did not alter rat body weight, glucose serum levels, triglycerides, total cholesterol, or hepatic enzyme activity (Table 4), suggesting that the particles not only avoided damaging the liver but also exhibited good biocompatibility.

**Table 4:** Basal metabolic parameters of HHTg animals untreated and treated with γ-Fe_2_O_3_/CeO_x_-based nanoparticles.

	**Untreated**	**γ-Fe_**2**_O_**3**_**	**γ-Fe_**2**_O_**3**_/CeO_**x**_**	**γ-Fe_**2**_O_**3**_/CeO_**x**_@PEG_**2, 000**_**	**γ-Fe_**2**_O_**3**_/CeO_**x**_@PEG_**5, 000**_**	***p***
Body weight (g)	405 ± 13	386 ± 46	387 ± 25	417 ± 36	408 ± 18	n.s.
Triacylglycerols (mmol/l)	3.22 ± 0.33	3.47 ± 0.20	3.45 ± 0.46	3.84 ± 0.34	2.96 ± 0.62	n.s.
Cholesterol (mmol/l)	1.88 ± 0.10	2.04 ± 0.35	1.86 ± 0.19	1.98 ± 0.10	1.85 ± 0.12	n.s.
Non-fasting glucose (mmol/l)	8.71 ± 0.22	8.05 ± 1.60	8.13 ± 0.38	8.70 ± 0.44	7.96 ± 0.60	n.s.
ALT (μkat/l)	1.16 ± 0.12	1.07 ± 0.08	1.09 ± 0.14	1.04 ± 0.06	1.19 ± 0.50	n.s.
AST (μkat/l)	2.75 ± 0.09	2.41 ± 0.11	2.70 ± 0.48	2.25 ± 0.18	2.76 ± 0.07	n.s.

*ALT, alanine aminotransferase; AST, aspartate aminotransferase; p, statistical significance; n.s., not significant*.

**Figure 7:**
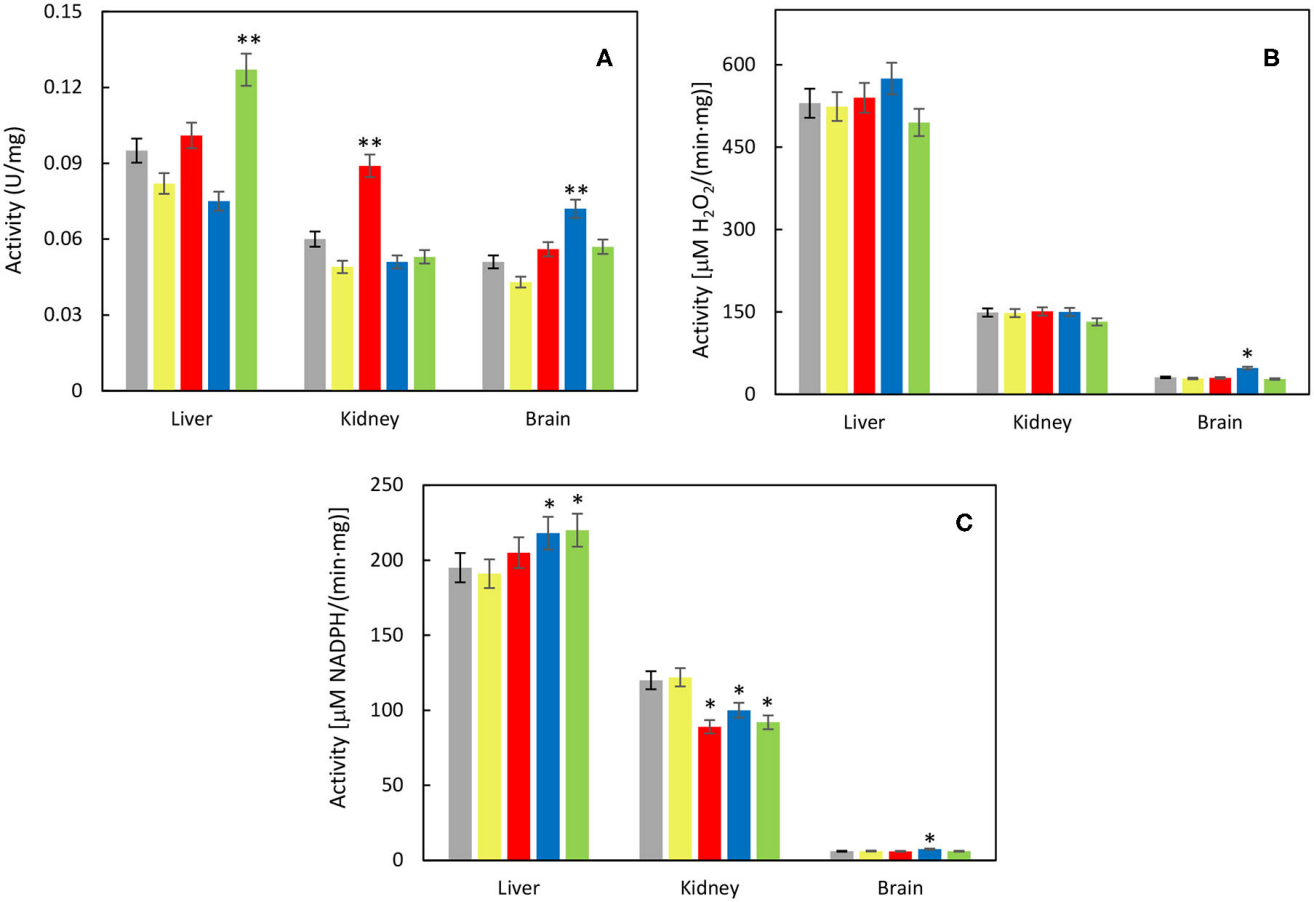
Effect of γ-Fe_2_O_3_ (yellow), γ-Fe_2_O_3_/CeO_x_ (red), γ-Fe_2_O_3_/CeO_x_@PEG_2,000_ (blue), and γ-Fe_2_O_3_/CeO_x_@PEG_5,000_ nanoparticles (green) on activity of **(A)** superoxide dismutase (SOD), **(B)** catalase (CAT), and **(C)** glutathione peroxidase (GPx) in the tissues of HHTg rats; rats untreated with nanoparticles are in gray. ^*^p < 0.05 and ^**^p < 0.01.

**Figure 8:**
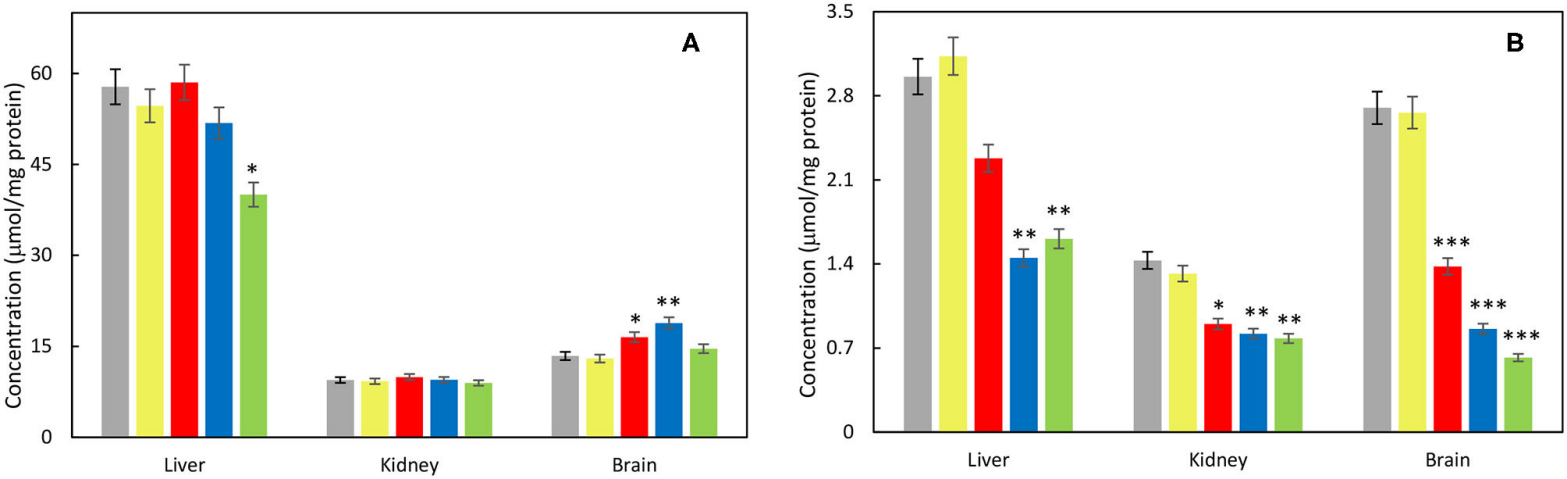
Effect of γ-Fe_2_O_3_ (yellow), γ-Fe_2_O_3_/CeO_x_ (red), γ-Fe_2_O_3_/CeO_x_@PEG_2,000_ (blue), and γ-Fe_2_O_3_/CeO_x_@PEG_5,000_ nanoparticles (green) on level of **(A)** reduced (GSH) and **(B)** oxidized forms of glutathione (GSSG) in the tissues of HHTg rats; rats untreated with nanoparticles are in gray. ^*^p < 0.05, ^**^p < 0.01, and ^***^p < 0.001.

**Figure 9:**
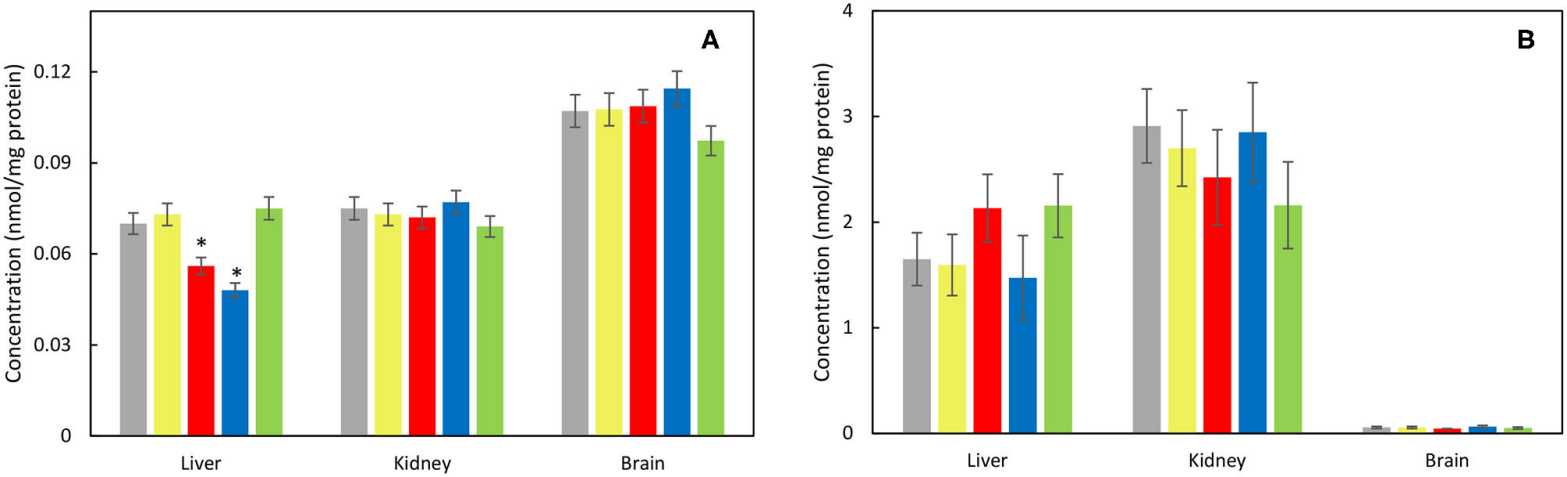
Effect of γ-Fe_2_O_3_ (yellow), γ-Fe_2_O_3_/CeO_x_ (red), γ-Fe_2_O_3_/CeO_x_@PEG_2,000_ (blue), and γ-Fe_2_O_3_/CeO_x_@PEG_5,000_ nanoparticles (green) on concentrations of **(A)** malondialdehyde (MDA) and **(B)** 4-hydroxynonenal (4-HNE) markers of lipid peroxidation in the tissues of HHTg rats; rats untreated with nanoparticles are in gray. ^*^p < 0.05.

Considering oxidative stress status, effect of each nanoparticle type was investigated on liver, kidney cortex, and brain tissues. The activity of antioxidant enzyme SOD increased significantly in the liver after γ-Fe_2_O_3_/CeO_x_@PEG_5,000_ administration as well as in the brain after γ-Fe_2_O_3_/CeO_x_@PEG_2,000_ and in the kidney after γ-Fe_2_O_3_/CeO_x_ exposure, compared to the animal control group treated with γ-Fe_2_O_3_ nanoparticles (Figure 7A). CAT activity increased after the administration of γ-Fe_2_O_3_/CeO_x_@PEG_2,000_ in the brain only (Figure 7B). After exposure of γ-Fe_2_O_3_/CeO_x_@PEG_2,000_, the activity of glutathione-dependent enzyme GPx increased in the liver and brain as well as in the liver after γ-Fe_2_O_3_/CeO_x_@PEG_5,000_ nanoparticles administration (Figure 7C). In the brain, elevated levels of the reduced form (GSH) of glutathione and decreased levels of the oxidized form (GSSG) after γ-Fe_2_O_3_/CeO_x_@PEG_2,000_ and γ-Fe_2_O_3_/CeO_x_ treatment improved oxidative status. The injection of γ-Fe_2_O_3_/CeO_x_@PEG_2,000_ and γ-Fe_2_O_3_/CeO_x_@PEG_5,000_ in HHTg rats also markedly decreased the oxidized form of glutathione in the liver and kidney cortex (Figures 8A,B). After γ-Fe_2_O_3_/CeO_x_@PEG_2,000_ particle administration, reduced levels of MDA were observed in the liver (Figure 9A). The concentration of another product of lipid peroxidation, 4-HNE, did not change after nanoparticle applications in the case of all tissues examined, indicating no oxidative damage to lipids (Figure 9B).

## Discussion

**Figure 10:**
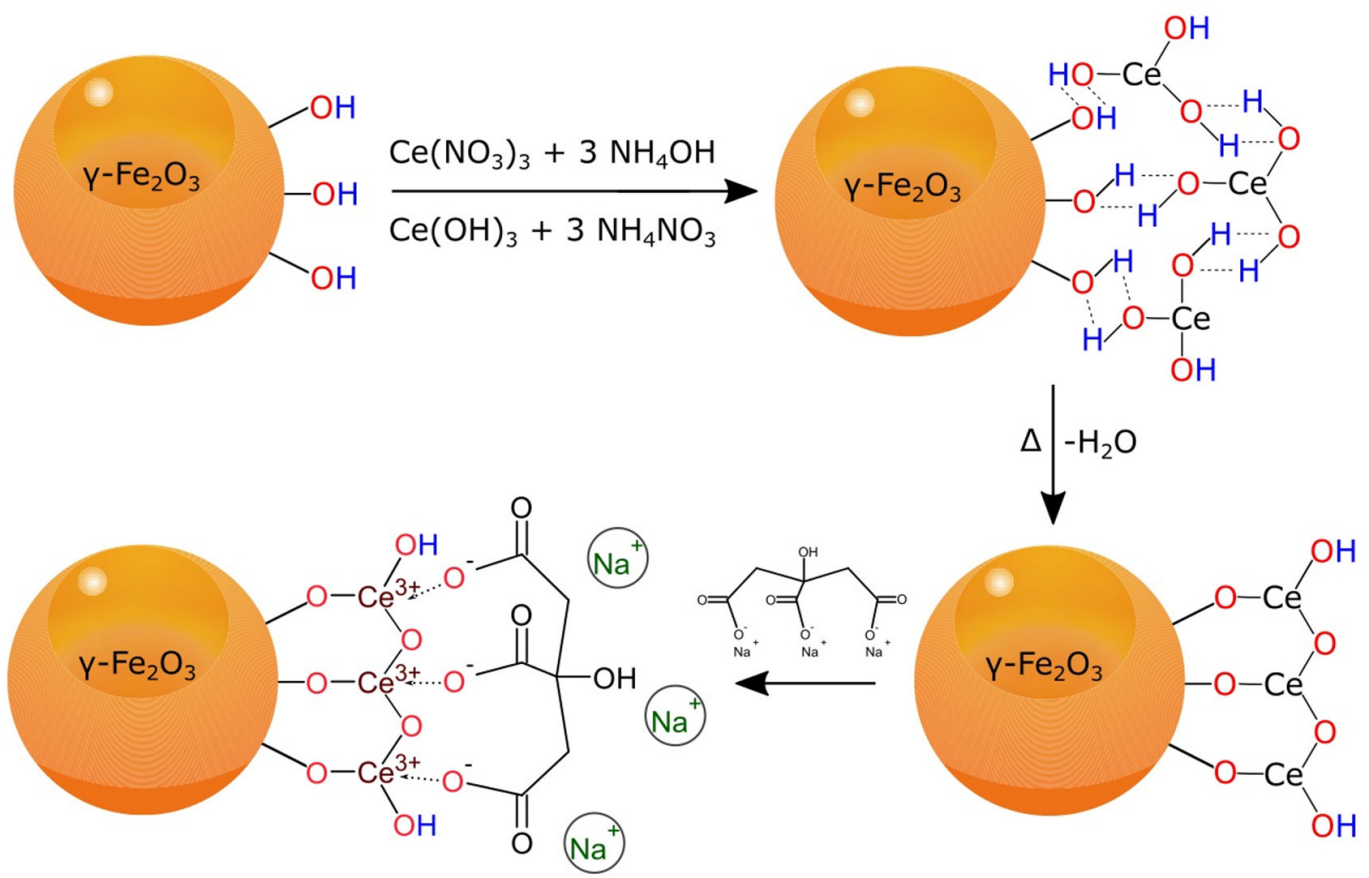
Synthesis of sodium citrate-stabilized γ-Fe_2_O_3_/CeO_x_ nanoparticles by dispersion precipitation of Ce(NO_3_)_3_ with NH_4_OH.

In order to obtain magnetic particles possessing antioxidant properties, γ-Fe_2_O_3_ particles were used as seeds for CeO_x_ growth (Figure 10). Dispersion precipitation of Ce(NO_3_)_3_ with ammonia (pH 11) resulted in the formation of Ce(OH)_3_ nuclei, which were attached to the γ-Fe_2_O_3_ surface by hydrogen bonds. This was followed by the conversion of Ce(OH)_3_ to Ce_2_O_3_ using a dehydration reaction under heating. The subsequent growth of cerium oxides was stabilized by sodium citrate, resulting in a ceria shell formation around the iron oxide core. XRD confirmed the presence of γ-Fe_2_O_3_ and CeO_2_ crystalline forms in the γ-Fe_2_O_3_/CeO_x_ particles. Further coating by PEG_2,000_ did not affect the crystalline structure of the γ-Fe_2_O_3_/CeO_x_ nanoparticles or induced crystallization of PEG. Based on a comparison of the high-resolution Ce 3d spectra (Figure 4) with data from the literature (Beche et al., 2008), we conclude that cerium in γ-Fe_2_O_3_/CeO_x_ nanoparticles coexisted in both Ce^3+^ and Ce^4+^ oxidation states, the latter being the most predominant. The saturation magnetization of starting γ-Fe_2_O_3_ was relatively comparable to previous reports (Lai et al., 2003; Zasonska et al., 2019), albeit lower than that of bulk maghemite. Because these particles are small in size, they are less magnetic than the same material in bulk, since they contain a higher fraction of metal ions on the particle surface that fail to contribute to net magnetization (Kucheryavy et al., 2013).

**Figure 11:**
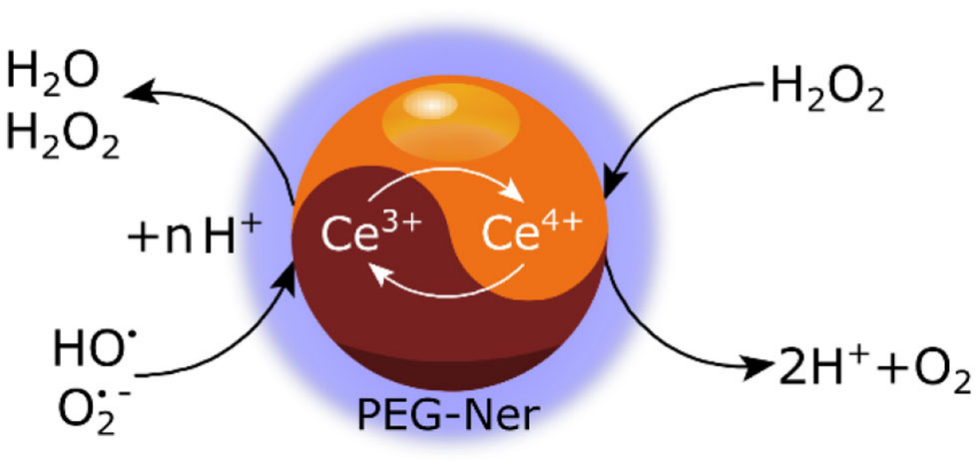
Schematic view of hydrogen peroxide and superoxide radical scavenging due to a Ce^3+^/Ce^4+^ redox shift on γ-Fe_2_O_3_/CeO_x_@PEG nanoparticles.

In order to stabilize the γ-Fe_2_O_3_/CeO_x_ nanoparticles in a culture medium and render them biocompatible, they must be coated with a polymer that is both amphiphilic and hydrophilic. PEG-Ner is an eminently suitable candidate, boasting extremely high affinity of bisphosphonate groups to metal cations (in our case Ce^3+^) deposited on the γ-Fe_2_O_3_ surface. This post-modification of γ-Fe_2_O_3_/CeO_x_ nanoparticles differs from one-pot synthesis of dextran-coated nanoceria, which involves the alkaline-based precipitation of cerium oxide from a solution containing cerium salt and dextran (Perez et al., 2008). One of the benefits of CeO_x_ is its ability to catalytically scavenge ROS, including H_2_O_2_, hydroxyl, and superoxide radicals due to the high number of surface cerium atoms capable of alternating between Ce^3+^ and Ce^4+^ (Figure 11). The further ability of CeO_x_ to self-regenerate its surface makes the γ-Fe_2_O_3_/CeO_x_@PEG nanoparticles an ideal candidate for *in vivo* ROS scavenging.

In this report, antioxidant activity of γ-Fe_2_O_3_, γ-Fe_2_O_3_/CeO_x_, and γ-Fe_2_O_3_/CeO_x_@PEG nanoparticles was documented by DPPH assay, a convenient and accurate method for chemically determining radical scavenging ability. γ-Fe_2_O_3_ itself exhibited relatively low *AA* due to the redox properties of Fe^3+^ ions and the large specific surface area of the particles. In contrast, the ability of cerium oxides attached to the γ-Fe_2_O_3_ surface to capture radicals was much higher than that of γ-Fe_2_O_3_. γ-Fe_2_O_3_/CeO_x_@PEG nanoparticles showed significantly higher antioxidant activity (*AA* ~90 %), compared to activity previously reported (*AA* ~45%) for poly(acrylic acid) (PAA)-stabilized Fe_3_O_4_/CeO_2_ core-shell nanoparticles determined by H_2_O_2_/horse radish peroxidase assay (Wu et al., 2018). Increased *AA* in γ-Fe_2_O_3_/CeO_x_@PEG particles can be induced by differences in the chemical structure and steric orientation of the above polymer coatings, thus restricting the accessibility of DPPH radicals to cerium ions in PAA-coated Fe_3_O_4_/CeO_2_ nanoparticles. This effect may also be partially due to differences in the assays employed.

To determine the antioxidant effect of cerium oxide-decorated magnetic particles, a strain of HHTg rats was deemed the most suitable experimental model given its tendency to exhibit increased oxidative stress in tissues (Malinska et al., 2019). We examined the tissues of the liver, kidney cortex, and brain, where metabolic disorders and changes are associated with metabolic syndrome and diabetes, after administering each nanoparticle type. Based on *in vivo* tests, Fe_2_O_3_/CeO_x_@PEG_2,000_ were found to exhibit a superior antioxidant defense to γ-Fe_2_O_3_/CeO_x_@PEG_5,000_ and γ-Fe_2_O_3_/CeO_x_ particles, activating most antioxidant enzymes and reducing the concentration of MDA formed by lipid peroxidation in the liver. Fe_2_O_3_/CeO_x_@PEG_2,000_ exhibited a positive effect on the brain by increasing GSH levels while simultaneously decreasing GSSG concentrations, thus improving oxidative status. Last but not least, Fe_2_O_3_/CeO_x_@PEG particles also demonstrated their capacity to circulate freely within the rat blood stream and thus reach and positively affect target tissues, an especially important asset in preventing the development of diabetic complications.

## Conclusion

We developed a combination of antioxidant CeO_x_ nanoparticles and superparamagnetic γ-Fe_2_O_3_ particles by dual coprecipitation and oxidation of Fe chlorides and Ce nitrate in alkaline media. The colloidal stability and biocompatibility of the resulting γ-Fe_2_O_3_/CeO_x_ particles was clearly boosted by PEGylation. PEG-neridronate proved an excellent stabilizing agent for preventing particle aggregation. γ-Fe_2_O_3_/CeO_x_ particles reached the target tissues and ameliorated oxidative stress in tissues. The PEG coating did not affect the autocatalytic properties of nanoceria, given that hydrogen peroxide and peroxyl radicals were able to diffuse through the hydrophilic PEG shell and oxidize Ce^3+^ to Ce^4+^. Representing another significant advantage, γ-Fe_2_O_3_/CeO_x_@PEG nanoparticles can be prospectively monitored by MRI, facilitating the tracking of cerium oxide delivery to the diseased site and the evaluation of ceria biodistribution. Combining the antioxidant defense properties of cerium oxide with a magnetic carrier thus provides great benefits for magnetic targeting. Compared to the administration of γ-Fe_2_O_3_/CeO_x_@PEG_5,000_, γ-Fe_2_O_3_/CeO_x_, and control γ-Fe_2_O_3_ nanoparticles, the effect of γ-Fe_2_O_3_/CeO_x_@PEG_2,000_ nanoparticles on parameters of oxidative stress in the brain and liver of HHTg rats was superior. The γ-Fe_2_O_3_/CeO_x_@PEG_2,000_ nanoparticles proved to positively influence oxidative stress in experimental animals, thus providing protection against oxidative damage. As such, the particles offer great potential for reducing complications associated with various diseases accompanied by increased oxidative stress such as cardiovascular disorders, diabetes mellitus, liver steatosis, and inflammatory conditions.

## Data Availability Statement

All datasets generated for this study are included in the article/supplementary material.

## Ethics Statement

The animal study was reviewed and approved by the Ethics Committee of the Institute for Clinical and Experimental Medicine, Prague, Czechia.

## Author Contributions

MM synthesized the particles. IM, HM, DM, MH, and OO performed ROS scavenging and *in vivo* studies of the particles. OP-G, AZ, and EP performed XPS, XRD, and magnetic measurements, respectively. DH supervised the work and together with IM wrote the publication. All authors contributed to the article and approved the submitted version.

## Conflict of Interest

The authors declare that the research was conducted in the absence of any commercial or financial relationships that could be construed as a potential conflict of interest.

## Footnotes

^1^http://www.crystallography.net/cod/9012692.html
